# Supplementary material for: Splice-Junction-Based Mapping of Alternative Isoforms in the Human Proteome
Source: Cell Rep. Author manuscript; Available in PMC 2020 Jan 15. (PMC6961840; doi:10.1016/j.celrep.2019.11.026)
Supplement: 2 [file NIHMS1546469-supplement-2.zip › DF1/PXD006675/RightAtrium/RightAtrium_37_TTN_INVLEPPK.pdf]

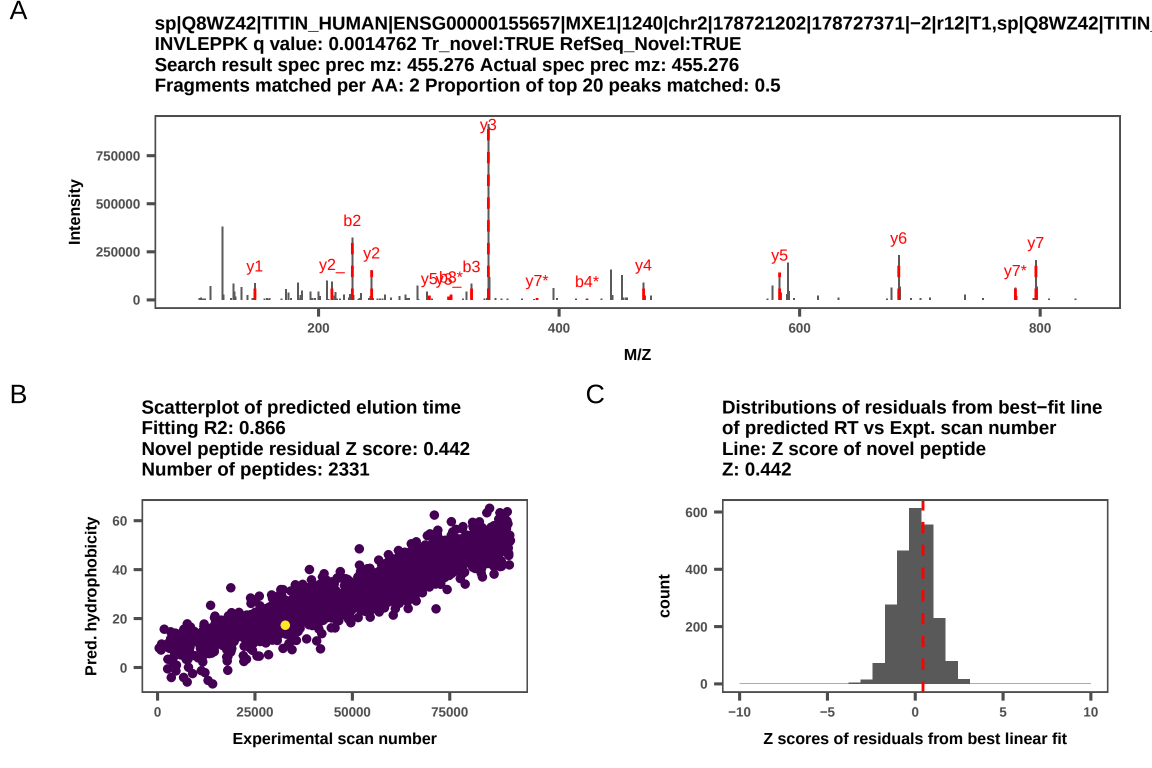
A
sp|Q8WZ42|TITIN_HUMAN|ENSG00000155657|MXE1|1240|chr2|178721202|178727371|−2|r12|T1,sp|Q8WZ42|TITIN_
INVLEPPK q value: 0.0014762 Tr_novel:TRUE RefSeq_Novel:TRUE
Search result spec prec mz: 455.276 Actual spec prec mz: 455.276
Fragments matched per AA: 2 Proportion of top 20 peaks matched: 0.5
Intensity
750000
500000
250000
0
y3
b2
y2
y1
y2_
y5
y8*
b3
y7*
b4*
y4
y5
y6
y7*
y7
200
400
600
800
M/Z
B
Scatterplot of predicted elution time
Fitting R2: 0.866
Novel peptide residual Z score: 0.442
Number of peptides: 2331
Pred. hydrophobicity
60
40
20
0
0
25000
50000
75000
Experimental scan number
C
Distributions of residuals from best−fit line
of predicted RT vs Expt. scan number
Line: Z score of novel peptide
Z: 0.442
count
600
400
200
0
−10
−5
0
5
10
Z scores of residuals from best linear fit
